# Whole Genome Sequencing Prioritizes *CHEK2, EWSR1*, and *TIAM1* as Possible Predisposition Genes for Familial Non-Medullary Thyroid Cancer

**DOI:** 10.3389/fendo.2021.600682

**Published:** 2021-02-22

**Authors:** Aayushi Srivastava, Sara Giangiobbe, Diamanto Skopelitou, Beiping Miao, Nagarajan Paramasivam, Chiara Diquigiovanni, Elena Bonora, Kari Hemminki, Asta Försti, Obul Reddy Bandapalli

**Affiliations:** ^1^ Division of Molecular Genetic Epidemiology, German Cancer Research Center, Heidelberg, Germany; ^2^ Preclinical Pediatric Oncology, Hopp Children’s Cancer Center (KiTZ), Heidelberg, Germany; ^3^ Division of Pediatric Neurooncology, German Cancer Research Center (DKFZ), German Cancer Consortium (DKTK), Heidelberg, Germany; ^4^ Medical Faculty, Heidelberg University, Heidelberg, Germany; ^5^ Computational Oncology, National Center for Tumor Diseases (NCT), Molecular Diagnostics Program, Heidelberg, Germany; ^6^ Unit of Medical Genetics, Department of Medical and Surgical Sciences, S. Orsola-Malphigi Hospital, University of Bologna, Bologna, Italy; ^7^ Faculty of Medicine and Biomedical Center in Pilsen, Charles University in Prague, Pilsen, Czechia

**Keywords:** *CHEK2*, *EWSR1*, *TIAM1*, familial non-medullary thyroid cancer, germline variant, non-syndromic, whole-genome sequencing

## Abstract

Familial inheritance in non-medullary thyroid cancer (NMTC) is an area that has yet to be adequately explored. Despite evidence suggesting strong familial clustering of non-syndromic NMTC, known variants still account for a very small percentage of the genetic burden. In a recent whole genome sequencing (WGS) study of five families with several NMTCs, we shortlisted promising variants with the help of our in-house developed Familial Cancer Variant Prioritization Pipeline (FCVPPv2). Here, we report potentially disease-causing variants in checkpoint kinase 2 (*CHEK2*), Ewing sarcoma breakpoint region 1 (*EWSR1*) and T-lymphoma invasion and metastasis-inducing protein 1 (*TIAM1*) in one family. Performing WGS on three cases, one probable case and one healthy individual in a family with familial NMTC left us with 112254 variants with a minor allele frequency of less than 0.1%, which was reduced by pedigree-based filtering to 6368. Application of the pipeline led to the prioritization of seven coding and nine non-coding variants from this family. The variant identified in *CHEK2*, a known tumor suppressor gene involved in DNA damage-induced DNA repair, cell cycle arrest, and apoptosis, has been previously identified as a germline variant in breast and prostate cancer and has been functionally validated by Roeb et al. in a yeast-based assay to have an intermediate effect on protein function. We thus hypothesized that this family may harbor additional disease-causing variants in other functionally related genes. We evaluated two further variants in *EWSR1* and *TIAM1* with promising in silico results and reported interaction in the DNA-damage repair pathway. Hence, we propose a polygenic mode of inheritance in this family. As familial NMTC is considered to be more aggressive than its sporadic counterpart, it is important to identify such susceptibility genes and their associated pathways. In this way, the advancement of personalized medicine in NMTC patients can be fostered. We also wish to reopen the discussion on monogenic vs polygenic inheritance in NMTC and instigate further development in this area of research.

## Introduction

Thyroid cancer (TC) is the most common endocrine malignancy with a global average age-standardized incidence of 6.7/100,000 persons per year (1). In 2018, 567,000 new cases were diagnosed, and 41,000 deaths were recorded, making it the tenth most frequently diagnosed cancer in that year (1). Despite these figures, the current knowledge on the genetic basis of non-medullary thyroid cancer (NMTC), an entity accounting for over 95% of all TC cases, is sparse (2).

Familial cases constitute about 5% to 15% of all NMTCs and can be subcategorized into syndromic and non-syndromic forms (2). Although syndromic forms are rare, germline susceptibility genes involved in each of the syndromes are known (3). The lack of knowledge surrounding the non-syndromic form is intriguing and has been previously pinned to the flawed nature of its definition since the inclusion of families with only two-affected member has led to the erroneous inclusion of many sporadic cases in such familial studies (4). Our study is based on a family with five affected members and we are confident that each of the cases represents a true familial case.

Albeit still highly controversial, some studies have suggested that the clinical characteristics of FNMTC are distinct from its sporadic counterpart, including earlier age of onset, a higher incidence of multifocality and a more aggressive form of the disease (5, 6). This insight reinforces the importance of identifying the genetic factors predisposing to this familial disease. In recent years, a plethora of approaches, including genome-wide association studies, linkage analyses, targeted sequencing, and whole exome sequencing, have been implemented in the hope of understanding FNMTC. Several genes and loci, including mainly low-penetrance variants near or in *FOXE1, SRGAP1, TITF-1/NKX2-1, DIRC3*, and *CHEK2*, have been suggested to affect non-syndromic FNMTC susceptibility (7, 8). An imbalance of the telomere-telomerase complex has also been reported in the peripheral blood of familial papillary thyroid cancer patients (9).

We performed whole genome sequencing (WGS) on five families with several family members diagnosed with NMTC in a recent study and shortlisted variants using our in-house familial cancer variant prioritization pipeline (FCVPPv2) in conjunction with other pathway and network analysis tools (10). In this study, we expand on these results by conducting further *in silico* analyses on the variants prioritized in a family and identifying three potentially disease-causing germline variants in *CHEK2*, *EWSR1*, and *TIAM1*. The CHEK2 variant we identified (p.E239K) has already been reported as a germline variant in breast and prostate cancers (11, 12). The variant has also been functionally validated in a yeast-based assay, which showed it to have an intermediate effect on CHEK2-mediated DNA damage response (11). The authors suggested that breast cancer cases with such intermediate-activity mutations may also harbor a second predisposing allele (11). We extended this hypothesis to polygenic risk and analyzed one NMTC family and evaluated the other top variants for functional effects with *in silico* tools. We also consulted literature to understand if and how these variants could work together in synergy to lead to the NMTC phenotype in the affected family members. We thus suggest a polygenic mode of inheritance in the NMTC family studied here.

## Materials and Methods

### Patients

An Italian family with NMTC aggregation was recruited at the S. Orsola-Malpighi Hospital, Unit of Medical Genetics in Bologna, Italy. WGS was performed for samples from three affected members, one unaffected member, and one possible carrier, for which all blood samples were collected with informed consent following ethical guidelines approved by “Comitato Etico Indipendente dell ‘Azienda Ospedaliero-Universitaria di Bologna, Policlinico S. Orsola-Malpighi (Bologna, Italy)” and “comité consultatif de protection des personnes dans la recherche biomédicale, Le centre de lutte contre le cancer Léon-Bérard (Lyon, France).” Genomic DNA was extracted from the blood using the QiAMP DNA Blood Mini kit following the manufacturer´s instructions.

### Whole Genome Sequencing

Available genomic DNA samples from five members of the studied NMTC family were subjected to WGS using Illumina-based small read sequencing. Resulting data were mapped to the human reference genome (assembly GRCh37 version hs37d5) using BWA mem (version 0.7.8) and duplicates were removed using biobambam (version 0.0.148) (13). Small nucleotide variants (SNVs) and InDels were called through joint calling on all samples from the family with Platypus (14). ANNOVAR, 1000 Genomes, dbSNP, and ExAC data were accessed to annotate all variants (15–18). We retained variants based on QUAL scores (>20) and coverage (>5×) that also passed all the Platypus internal filters. We selected rare variants by selecting and removing variants with minor allele frequencies (MAFs) greater than 0.1% in the 1,000 Genomes Phase 3 and non-TCGA ExAC data. Pairwise comparison of shared rare variants was performed to check for sample swaps and family relatedness.

### Analysis and Prioritization of Coding Variants

We filtered and prioritized variants according to the criteria of our in-house developed variant prioritization pipeline FCVPPv2 (19). Variants were first selected based on pedigree data considering cancer patients as cases, individuals with benign nodules as potential mutation carriers and unaffected persons as controls. Variants were required to be present in all cases and none of the controls.

We then selected variants belonging to the top 10% of probable deleterious variants in the human genome, i.e. variants with Combined Annotation Dependent Depletion (CADD) scores greater than 10 using the web-based CADD tool v1.3 (20). Three separate scores, namely Genomic Evolutionary Rate Profiling (GERP), PhastCons, and PhyloP were used to evaluate the evolutionary conservation of the genomic position of a particular variant (21–23). GERP scores, PhastCons scores, and PhyloP scores were indicative of a good level of conservation and were thus used as thresholds in the selection of potentially causative variants. Variants predicted to be deleterious by at least 60% of the following tools accessed using dbNSFP namely SIFT, PolyPhen V2-HDV, PolyPhen V2-HVAR, LRT, MutationTaster, Mutation Assessor, FATHMM, MetaSVM, MetLR, and PROVEAN were retained for further analysis (24).

Next, we evaluated the intolerance of genes to functional mutations using scores derived from NHLBI-ESP6500, ExAC and a local data set, all of which were developed with allele frequency data (25). Two additional scoring systems developed by the ExAC consortium with the help of large-scale exome sequencing data were regarded for loss-of-function variants (pLI) and missense and synonymous variants (Z-scores). These were used for nonsense and missense variants, respectively. Intolerance scores were used to rank variants, rather than as cut-offs for selection.

These steps led to a final shortlist of variants, which was reviewed further with the help of published literature. We checked whether coding variants in important oncogenes, tumor suppressor genes or autosomal dominant familial syndrome genes had been missed by the cut-offs of the pipeline. These variants were handled leniently in the conservation and deleteriousness cut-offs and were included in the further analysis. Non-coding exonic variants were analyzed separately. Details of this analysis can be perused in our previous study (10).

### Selection and Validation of Candidate Variants

After filtering the variants based on the FCVPPv2, we visually inspected the WGS data for validity using the Integrative Genomics Viewer (IGV) (26). The final selection of potentially causative variants was based on a thorough review of available literature. We screened WGS data from the other four NMTC families that were part of our previous study for the selected variants. Selected variants were validated by Sanger sequencing of DNA samples of all available family members using specific primers for polymerase chain reaction amplification designed with Primer3 (http://bioinfo.ut.ee/primer3-0.4.0/). Primer details are available on request. Sequencing was performed on a 3500 Dx Genetic Analyzer (Life Technologies, CA, USA) using ABI PRISM 3.1 Big Dye terminator chemistry according to the manufacturer’s instructions. The electrophoretic profiles were analyzed manually. Segregation of the variant with the disease was confirmed.

### Further *In Silico* Studies, Protein Alignment, and Structural Modeling

To further assess conservation of the shortlisted variants, ortholog gene groups for *CHEK2*, *TIAM1*, and *EWSR1* were obtained from the National Center for Biotechnology Information (NCBI). Multiple homologous sequences were submitted to and aligned by COBALT, a constraint-based multiple alignment tool (27). The NCBI accessions of the respective genes and their orthologs are detailed in **Table S1**. Alignments were visualized by COBALT and formatted manually.

Intolerance of the proteins to amino acid substitution was evaluated with the help of SNAP2, a neural network-based classifier (28). A heat map representation of independent substitutions for each position of the analyzed proteins (CHEK2, TIAM1, and EWSR1) was generated based on their tolerance to amino acid substitution. The amino acid position corresponding to the shortlisted variant being studied, as well as its surrounding region, was evaluated.

We assessed stability changes caused by the shortlisted variants for their respective proteins using the mutation Cutoff Scanning Matrix (mCSM) tool, provided that PDB structures containing the variant of interest were available for the protein (29). We accessed the PDB entry and 3D representation of human CHEK2 (2CN8) and human TIAM1 (1FOE) from the Protein Data Bank in Europe (30).

### Network Analysis

The proteins corresponding to the shortlisted variants (**Table S2**) were used as input for the STRING v11 network analysis (31). The first shell of interactors of the query proteins was included in the network. Network edge meaning was set to “evidence” and all active interaction sources were selected. The minimum required interaction score was set to medium confidence (0.400). The maximum number of interactors to show in the first shell was set to 10. Disconnected nodes of the network were hidden.

## Results

### Whole Genome Sequencing and Variant Prioritization

**Figure 1:**
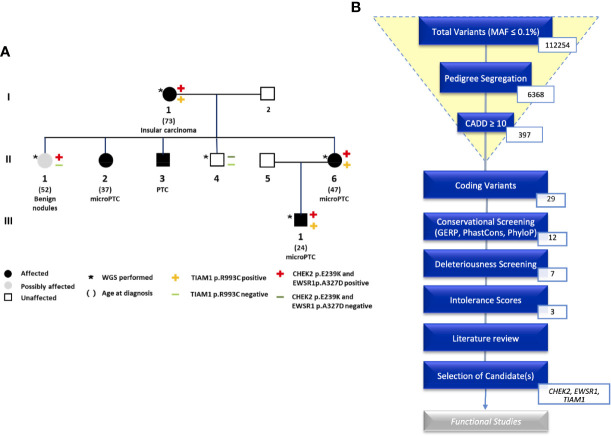
**(A)** Pedigree of the NMTC family with *CHEK2*, *TIAM1*, and *EWSR1* mutations. **(B)** An overview of the analysis of coding variants following the Familial Cancer Variant Prioritization Pipeline version 2 (FCVPPv2).

This study is based on an Italian family with five family members diagnosed with NMTC (**Figure 1A**). Four family members are affected by PTC or micro-PTC (II-2, II-3, II-6, III-1), one is affected by insular carcinoma (I-1), one is a possible carrier with benign nodules (II-1), and one is unaffected (II-4). We obtained samples from five of these family members for WGS. The variants were filtered based on pedigree data considering family members diagnosed with NMTC or micro-PTC as cases, benign nodules as potential variant carriers, and unaffected members as controls.

WGS identified a total of 112254 variants with minor allele frequency of less than 0.1%. This number was reduced by pedigree-based filtering to 6368. Of these variants, seven non-synonymous coding variants and nine non-coding variants segregated with the disease and passed the filters of our variant prioritization pipeline (10). Intolerance scores were then used to rank variants. No nonsense mutations made it to the shortlist. We were left with three variants in the following genes: *CHEK2, TIAM1*, and *EWSR1*. We screened available WGS data from the other four NMTC families that were part of our previous study for these three variants. These were not present in any of the four families.

All shortlisted variants with their scores can be viewed in the supplementary data (**Table S2**). An overview of the prioritization process is shown in **Figure 1B**.

### *In Silico* Studies Show Functional Importance of Mutations in CHEK2, EWSR1, and TIAM1

**Figure 2:**
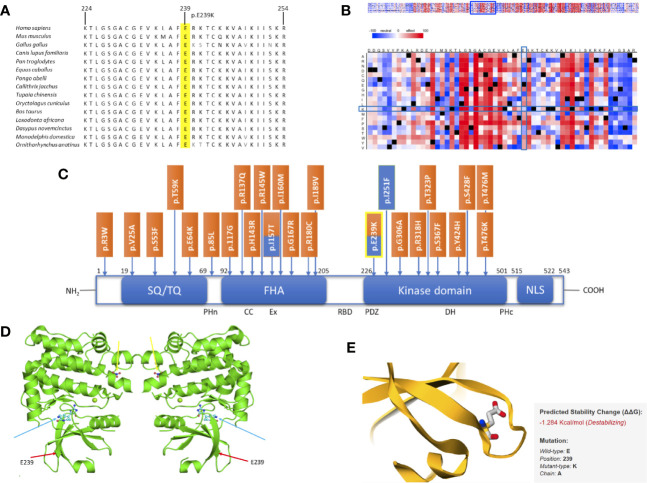
*In silico* studies of CHEK2 p. E239K. **(A)** Comparative sequence analysis across representative phylogeny. The mutation site is highlighted in yellow. **(B)** SNAP2 results. **(C)** Schematic diagram of the CHEK2 protein with all domains, listed from the N-terminal to the C-terminal, modified and adapted from Roeb et al.: SQ/TQ-rich domain (residues 19–69), forkhead-associated (FHA) domain (residues 92–205) and kinase domain (residues 226–501). Known germline variants in breast cancer (orange) and prostate cancer (blue) are shown according to their positions. The variant identified in this study is highlighted in yellow. **(D)** Dimeric assembly of PDB entry 2CN8 colored by chemically distinct molecules and viewed from the front. Debromohymenialdisine molecules are represented as ball-and-stick structures (blue arrows). Magnesium ions are shown as small green spheres. Nitrate ions are also represented as ball-and-stick structures (yellow arrows). The position of our variant is marked using red arrows. **(E)** Thermodynamic change in Gibb’s free energy caused by the p.E239K substitution as predicted by the mutation Cutoff Scanning Matrix (mCSM).

Comparative sequence analysis of *CHEK2* showed our position (E239K) and the region surrounding it to be highly conserved among selected representative species within the phylogeny (**Figure 2A**). The tolerance of amino acid substitutions at and around position 239 of the CHEK2 protein was predicted by SNAP2 and presented as a heat-map. The glutamate to lysine substitution is shown to have a neutral effect (−20; **Figure 2B**). The region immediately preceding this position, spanning a length of about twelve amino acids, is shown in the heat-map to be highly intolerant to substitutions. The variant identified in this study is located in the kinase domain of the CHEK2 protein. It has previously been reported as a germline variant in both, breast and prostate cancers. Known germline variants in *CHEK2* in breast and prostate cancers span the length of the protein, with aggregation in the forkhead-associated and kinase domains and are shown in **Figure 2C** (11, 32). The dimeric assembly of the crystal structure of human CHEK2 in complex with debromohymenialdisine was attained from the Protein Data Bank in Europe and is shown in **Figure 2D** (33). We used this entry as input for the mutation Cutoff Scanning Matrix (mCSM) approach to predict protein stability. The mCSM approach depends on graph-based signatures to predict the impact of missense mutations on protein stability. The thermodynamic change in free energy caused by the p.E239K mutation was predicted to be destabilizing (ΔΔG = −1.284 Kcal/mol, **Figure 2E**).

**Figure 3:**
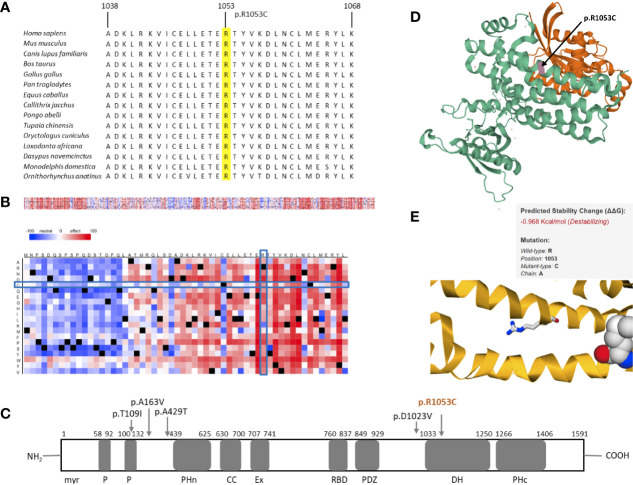
*In silico* studies of TIAM1 p.R1053C. **(A)** Comparative sequence analysis across representative phylogeny. The position is highlighted in yellow. **(B)** Heat map representation of SNAP2 results depicting the likely impact of individual amino acid substitutions (y-axis) for each position (x-axis) on protein function. Substitutions with strong effects are depicted with dark red (score = 100), whereas weak substitutions are represented with blue (score = −100). White represents neutral substitutions. Black represents the corresponding wild type residue (upper panel). The R1053C position is shown with blue rectangles. **(C)** Schematic primary structure of the TIAM1 protein with its protein interaction domains adapted and modified from Boisser et al. TIAM1 contains the following domains, listed from the N terminus to the C terminus; myr, myristylation sequence; P, PEST domain; PHn, N-terminal PH domain; CC, coiled-coil region; Ex, extended domain; RBD, Ras binding domain; PDZ, PSD-95/DlgA/ZO-1 domain; DH, Dby homology domain; PHc, C-terminal PH domain. Germline variants in TIAM1 identified thus far are shown with arrows. The variant identified in this study is colored orange. **(D)** 3D crystal structure of PDB 1FOE, which contains 1 copy of T-lymphoma invasion and metastasis-inducing protein 1 (TIAM1, green), 1 copy of Ras-related C3 botulinum toxin substrate 1 (orange), and 1 copy of sulfate ion in assembly 1. TIAM1 is colored in green. R1053C is highlighted in magenta and marked with an arrow. **(E)** Thermodynamic change in Gibb’s free energy caused by the p.R1053C substitution as predicted by the mutation Cutoff Scanning Matrix (mCSM).

Similarly, the second protein we analyzed further showed promising results. Multiple sequence alignment of amino acids 1038–1068 of *TIAM1* from selected vertebrate species indicated the region surrounding the identified variant to be highly conserved (**Figure 3A**). The SNAP2 heat-map representation showed a deleterious effect (score = 41) of almost all substitutions in position 1053. The position’s imminent surrounding region is also shown to be intolerant to amino acid substitutions, which is put into stark contrast with predominantly neutral region 24 amino acids upstream of the variant (**Figure 3B**). The variant identified in this study (p.R1053C) is located in the Dbl homology domain (DH), which along with the C-terminal PH (PHc) domain, makes up the minimal unit required for nucleotide exchange (**Figure 3C**) (34). The PHc domain has been reported to play a role in TIAM1 localization and activation by way of direct regulation of catalytic activity of its associated-DH (35). Although *TIAM1* appears to promote cellular migration in colorectal carcinoma, gastric cancer, osteosarcoma, and ovarian cancer, the only *TIAM1* germline mutations that have been identified thus far are protective mutations in primary neuroblastomas. These are shown in **Figure 3C** (36–40).

The 3D crystal structure of the PDB entry 1FOE is shown in **Figure 3D** (41). The variant identified in this paper corresponds to residue 23 of the A chain of the PDB structure, which is in the DH domain of TIAM1. The DH domain (residues 1,034–1,258) is an elongated helical bundle composed of nine α-helices and four 3_10_-helices (41). Our position is located in one of the α-helices. We used this PDB entry for the mCSM analysis, which showed our mutation to cause destabilization of the TIAM1 protein, by predicting a negative thermodynamic change in Gibb’s free energy (ΔΔG = −0.968 Kcal/mol, **Figure 3E**).

**Figure 4:**
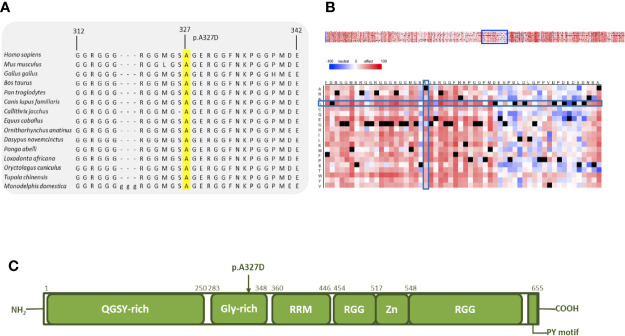
*In silico* analysis of EWSR1 p.A327D. **(A)** Comparative sequence analysis across representative phylogeny. The p.A327D position is highlighted in yellow. **(B)** Heat map representation of SNAP2 results depicting the likely impact of individual amino acid substitutions (y-axis) for each position (x-axis) on protein function. Substitutions with strong effects are depicted with dark red (score = 100), whereas weak substitutions are represented with blue (score = −100). White represents neutral substitutions. Black represents the corresponding wild type residue (upper panel). The A327D position is shown with blue rectangles. **(C)** Schematic primary structure of the EWSR1 protein with its domains adapted and modified from Couthouis et al.

We also obtained strong results for the final protein that was examined further with *in silico* tools. Comparative sequence analysis of amino acids 312–342 of *EWSR1* from selected representative species within the phylogeny shows the mutation site and the residues surrounding it to be highly conserved (**Figure 4A**). The heat-map representation of the SNAP2 results shows the substitutions at the site of the mutation (327) to have a deleterious effect (score, −54, **Figure 4B**). Again, the surrounding positions, excluding position 326, are predicted to be intolerant to amino acid substitutions. The schematic structure of EWSR1 with its domains is shown in **Figure 4C** (42). The protein consists of an N-terminal transcriptional-activation domain and a C-terminal nucleic acid binding domain. So far, no germline variants predisposing to cancer have been located in EWSR1. The variant identified in this study is in the glycine-rich domain, which, in fusion proteins, is responsible for the potent and oncogenic activation of a transcription factor fusion partner (43).

### Network Analysis Suggests Interaction of Top Three Variants

**Figure 5:**
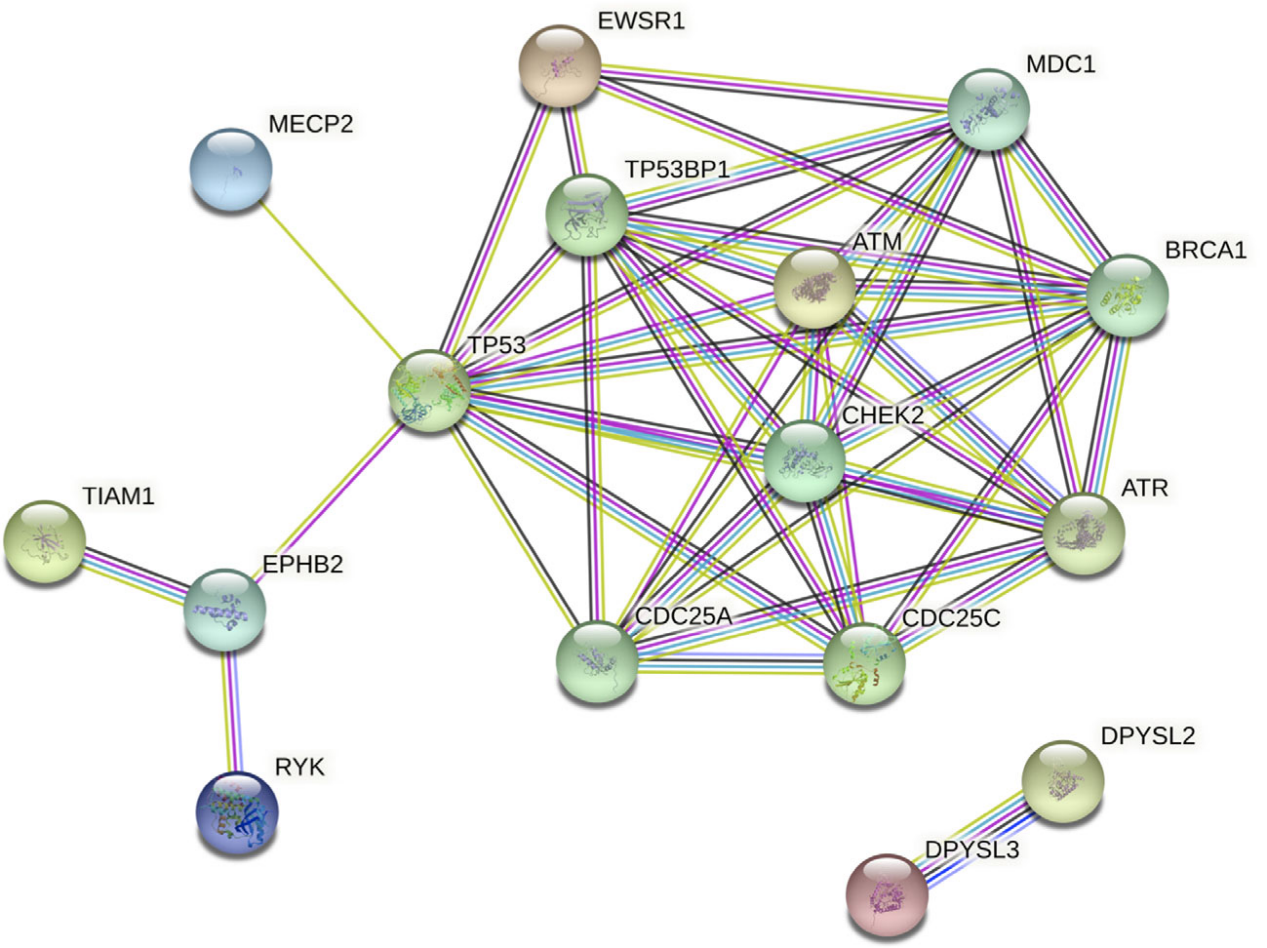
Protein interaction network of shortlisted genes generated by STRING v11. Intermediate regulators are added by STRING. Edges represent protein-protein associations. The type or source of the association can be interpreted using the line colors as follows: cyan, from curated databases (known); magenta, experimentally determined (known); green, gene neighborhood (predicted); red, gene fusions (predicted); dark blue, gene co-occurrence (predicted); green-yellow, text-mining; black, co-expression; purple, protein homology. Colored nodes represent query proteins and the first shell of interactors.

Network analysis *via* STRING showed clustering around TP53, CHEK2, and ATM. Intermediate regulators were added by STRING to include paths with more than one link. This enabled the generation of a comprehensive picture of possible gene interactions. These results indicate that the top deleterious variants of this family overwhelmingly target pathways related to cell-cycle regulation and DNA-damage repair (**Figure 5**). The three genes that were studied further with *in silico* tools interact in this network and play key roles in the aforementioned pathways, thus strengthening the case for a combined effect of all three genes (EWSR1, CHEK2, and TIAM1) as the driving force in the development of FNMTC in this family.

## Discussion

Although substantial advancements have been made in the field of genetics, with the emergence of WGS as a state-of-the-art tool in the identification of novel cancer-predisposing genes in Mendelian diseases, the genetic basis of FNMTC remains largely unexplored. A consensus as to the mode of inheritance, whether monogenic or polygenic, has yet to be reached. The inability of the scientific community to pinpoint one gene responsible for a large number of familial cases suggests a polygenic mode of inheritance, at least for a certain proportion of the familial cases. However, most published pedigrees, including some from our studies, are consistent with the inheritance of one autosomal dominant gene with reduced penetrance, supporting the theory of a monogenic mode of inheritance.

In this study, we prioritized three potentially deleterious coding variants in three genes, namely *CHEK2*, *TIAM1*, and *EWSR1* in an NMTC family with aggregation of PTC, micro-PTC, and insular carcinoma. All three variants showed promising results regarding their deleteriousness, with the exception of the SNAP2 results of *CHEK2*. The CHEK2 amino acid substitution was predicted to have only a neutral effect. However, given that the *CHEK2 p.E239K* variant is known in breast and prostate cancer and has been shown to have an intermediate effect on protein function in functional yeast-based studies, and that *TIAM1 p.R1053C* and *EWSR1 p.A327D* showed promising results in the *in silico* studies, we proceeded to explore the possibility of all three genes targeting functionally related pathways, thus acting as combined drivers of carcinogenesis in the studied NMTC family.

CHEK2 and EWSR1 interact in the DNA-damage repair pathway. CHEK2 is activated upon DNA damage and plays a key role in the induction of cell-cycle arrest. EWSR1 regulates alternative splicing and gene expression for genes involved in DNA damage response, cell growth, and apoptosis. In a study by Paronetto et al., knockdown of the Ewing sarcoma protein (EWS) led to higher levels of *CHEK2* exon 2 skipping, which in turn reduced levels of CHEK2 protein expression. EWS knockdown also led to reduced expression of the functional c-ABL protein, which is involved in the activation of p53 and p73, as well as to the formation of an isoform of MAP4K2 with reduced functionality (44, 45). MAP4K2, like CHEK2, is a serine/threonine kinase involved in DNA-damage response. Therefore, EWSR1 can affect genes important for cellular response to stress and DNA damage, such as *ABL1*, *CHEK2*, and *MAP4K2* (44).

TIAM1, although not in direct interaction with CHEK2 or EWSR1, is also a key player in the DNA-damage response of cells. CK1/β-TrCP-dependent TIAM1 destabilization is abolished in response to DNA damage and it accumulates in the cytoplasm. Accumulated TIAM1 contributes to apoptotic cell death by stimulating the Rac1/JNK cascade. Therefore, all three of the prioritized genes are involved in DNA-damage repair and cell-cycle regulation, which are central pathways in the prevention of tumorigenic changes within cells. It is known that ionizing radiation directly affects the risk of differentiated thyroid and breast cancers by inducing DNA double-strand breaks in exposed cells. As a consequence, the DNA repair pathway gains importance in patients exposed to such radiation. If genes involved in these pathways are affected by mutations, the likelihood of developing NMTC in response to exposure increases substantially. Moreover, such mutations could also predispose to cancer in the absence of ionizing radiation as the proper functionality of cell-cycle signaling, DNA repair, and DNA damage-induced apoptosis is imperative in the prevention of tumorigenesis.

There are, however, weaknesses in the hypothesis of the three genes having a synergistic effect on cancer development in the affected members of the family. Although *TIAM1* is linked to metastasis of colorectal cancer and thus to a more aggressive form of the disease, some studies show improvement in clinical outcome by germline *TIAM1* mutations in primary neuroblastoma (39). Further functional studies will be required to determine if the variants identified in our study represent bona fide pathogenic disease mutations for familial NMTC and to test if these mutations truly work in synergy. Furthermore, there is a possibility that the variants identified in this study are merely private mutations and thus not involved in the generic development of FNMTC. Sequencing of large cohorts will be required to address this question.

Although our study is not devoid of limitations, the most considerable being the lack of functional studies, the results of the *in silico* analyses described here will empower the evaluation of other *CHEK2, TIAM1*, and EWSR1 variants for potential pathogenicity and will reopen the debate regarding the mode of inheritance in FNMTC. Nevertheless, it is imperative that we gain a better understanding of the hereditary factors contributing to FNMTC susceptibility to identify aggressive cases while avoiding overdiagnosis.

In conclusion, WGS allowed us to identify three potentially disease-causing germline variants in *CHEK2, TIAM1*, and *EWSR1* in an NMTC-prone family. Furthermore, we propose a synergistic model of disease progression, as all three genes are part of cell-cycle regulation and DNA damage repair pathways, thereby suggesting a polygenic mode of inheritance of FNMTC in this family. Although *CHEK2* p. E239K has been identified in cancers of the breast and prostate, we are the first to report this variant in NMTC. *TIAM1* and *EWSR1* germline variants have also not been reported in FNMTC thus far. With such results, we hope to expand on the current knowledge regarding genetic factors leading to FNMTC and instigate further research focused on families with recurrence of non-syndromic NMTC. By these means, screening of families and other individuals at risk of developing NMTC can be improved substantially.

## Data Availability Statement

Unfortunately, for reasons of ethics and patient confidentiality, we are not able to provide the sequencing data into a public database. The data underlying the results presented in the study are available from the corresponding author or from AF (Email: a.foersti@kitz-heidelberg.de).

## Ethics Statement

The studies involving human participants were reviewed and approved by the committee for protection of persons in biomedical research of Lyon (CCPRB A-96.18) and by the IARC Ethical Review Board (Project 95-050, amendment 01-013). The patients/participants provided their written informed consent to participate in this study.

## Author Contributions

Conceptualization, KH, AF, and OB. Data curation, AS, NP, and OB. Formal analysis, NP, AS, and OB. Funding acquisition, KH. Investigation, AS and OB. Methodology, AS, DS, BM, and EB. Project administration, KH, AF, and OB. Resources, EB. Software, NP. Supervision, OB. Validation, EB. Writing-original draft, AS and OB. Review and editing, EB, KH, AF, and OB. All authors contributed to the article and approved the submitted version.

## Funding

KH was supported by the EU Horizon 2020 program grant no. 856620.

## Conflict of Interest

The authors declare that the research was conducted in the absence of any commercial or financial relationships that could be construed as a potential conflict of interest.

## Supplementary Material

Supplementary Table 1 Species used in the conservation analysis with their respective NCBI accession numbers for each gene.

Supplementary Table 2 Coding and non-coding variants with scores.
