# Supplementary material for: Whole Genome Sequencing Prioritizes CHEK2, EWSR1, and TIAM1 as Possible Predisposition Genes for Familial Non-Medullary Thyroid Cancer
Source: Front Endocrinol (Lausanne). 2021 Feb 22;12:600682. doi: 10.3389/fendo.2021.600682 (PMC7937922; doi:10.3389/fendo.2021.600682)
Supplement: Supplementary Table 1 — Species used in the conservation analysis with their respective NCBI accession numbers for each gene. [file Table_1.docx]

Table S1. Species used in the conservation analysis with their respective NCBI accession numbers for each gene.

| Species | Common Name | NCBI Accession Numbers (National Center for Biotechnology Information) | | |
| --- | --- | --- | --- | --- |
|  |  | CHEK2 | TIAM1 | EWSR1 |
| *Homo sapiens* | Human | NP_009125.1 | NP_001340618.1 | NP_005234.1 |
| *Mus musculus* | Mouse | XP_030110519.1 | NP_033410.2 | XP_006514571.1 |
| *Gallus gallus* | Chicken | XP_015130828.1 | XP_004938438.1 | XP_015150339.1 |
| *Canis lupus familiaris* | Dog | XP_005636448.2 | XP_544855.2 | XP_022265863.1 |
| *Pan troglodytes* | Chimpanzee | XP_016794370.1 | XP_531420.2 | XP_016794350.1 |
| *Equus caballus* | Horse | XP_001499632.1 | XP_001498883.1 | XP_023502669.1 |
| *Pongo abelli* | Orangutan | XP_024095726.1 | XP_002830672.2 | XP_002831024.1 |
| *Callithrix jacchus* | Marmoset | XP_009006835.1 | XP_017822589.1 | XP_009007040.1 |
| *Tupaia chinensis* | Tree Shrew | XP_027627394.1 | XP_006155324.1 | XP_006147954.1 |
| *Oryctolagus cuniculus* | Rabbit | XP_017206175.1 | XP_002716851.1 | XP_008272853.2 |
| *Bos taurus* | Cattle | NP_001029703.1 | XP_024849523.1 | XP_005218132.1 |
| *Loxodonta africana* | Elephant | XP_003419208.1 | XP_010588624.1 | XP_023415028.1 |
| *Dasypus novemcinctus* | Armadillo | XP_004461763.1 | XP_004468665.1 | XP_023445520.1 |
| *Monodelphis domestica* | Opossum | XP_016288010.1 | XP_007493453.1 | XP_001364505.1 |
| *Ornithorhynchus anatinus* | Platypus | XP_028904984.1 | XP_028938128.1 | XP_028904560.1 |
